# Supplementary material for: Concentric regulatory zones failed to halt surging COVID-19: Brooklyn 2020
Source: Front Public Health. 2022 Dec 7;10:970363. doi: 10.3389/fpubh.2022.970363 (PMC9768182; doi:10.3389/fpubh.2022.970363)
Supplement: Supplementary file 1 [file Data_Sheet_1.pdf]

Supplement to: Concentric Regulatory Zones Failed to Halt Surging COVID-19: Brooklyn 2020

Appendix A

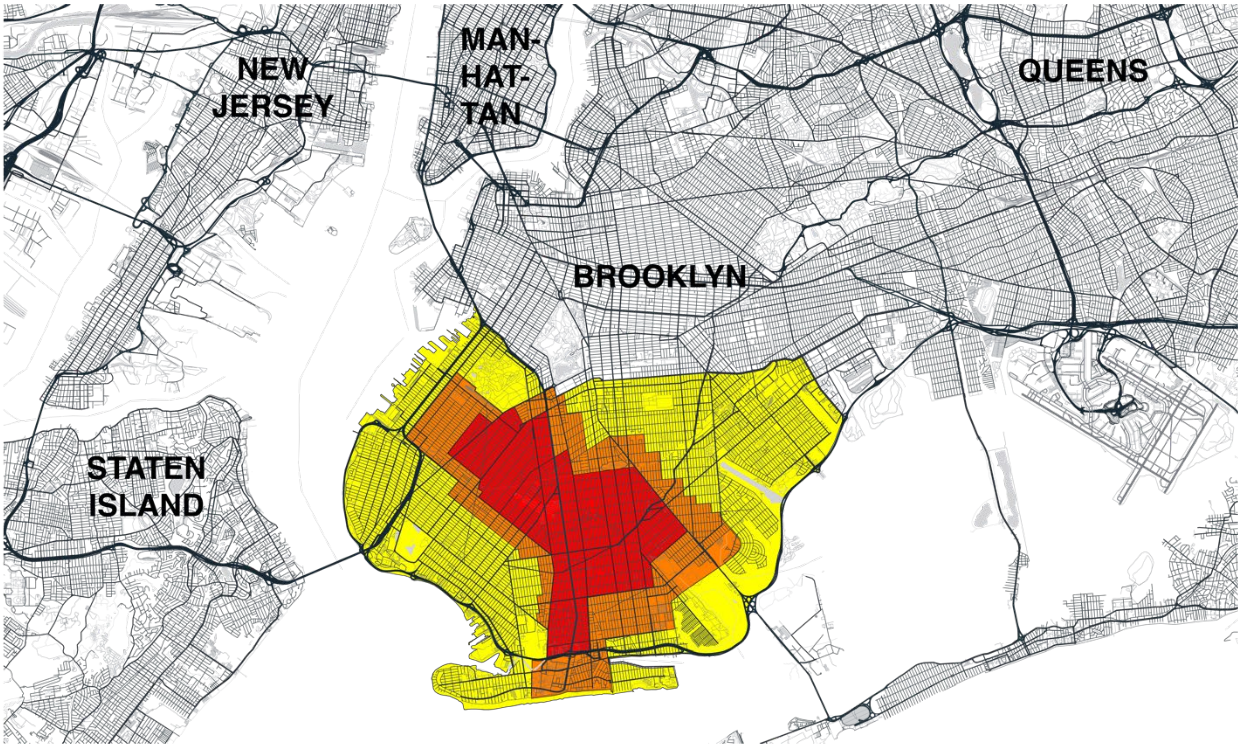

Fig A1. Concentric Regulatory Zones Established in South Brooklyn, Overlaid on Street Map of the New York City Area. The original three zones, color-coded red, orange, and yellow, went into effect on October 9, 2020 [1]. On October 21, the orange zone was redesignated as a yellow zone, while the red zone remained unchanged [2]. The entire regulated area, situated in South Brooklyn, was bounded along the north by New York State Route 27, originating in the northwest at exit 24 of Interstate I-278, with stretches along Prospect Parkway, Caton Avenue, Linden Boulevard, and then further bounded by Pennsylvania Avenue at the northeast end.

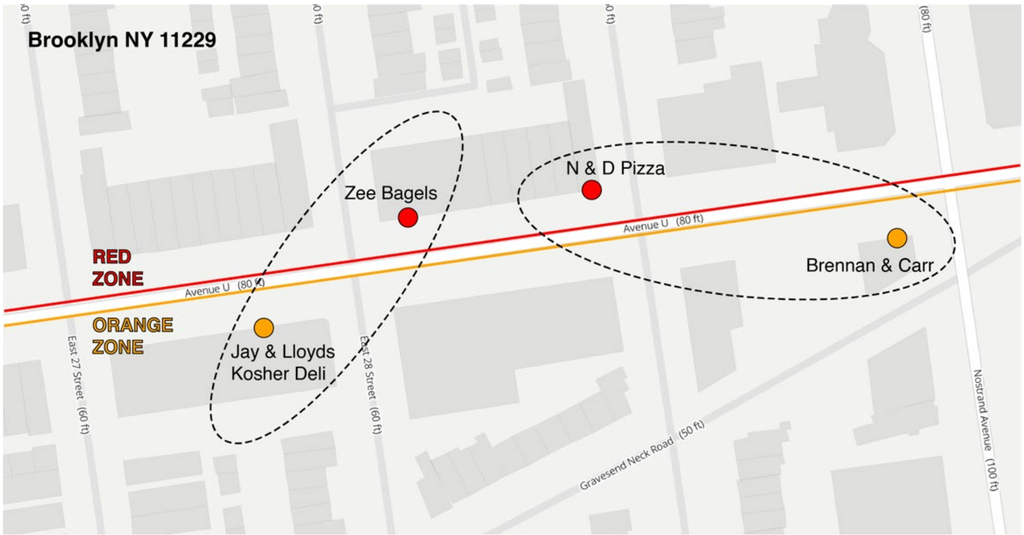

Fig. A2. Illustrative Paired Points of Interest Overlapping the Boundary Between the Original Red and Orange Zones. Avenue U bounds the red zone to the north and the orange zone to the south. *Zee Bagels* in the red zone was paired with *Jay & Lloyds Kosher Deli* (now permanently closed) in the orange zone. The distance between the two POIs was 61 meters. Similarly, *N & D Pizza* in the red zone was paired with *Brennan & Carr* in the orange zone. The distance between the two POIs was 92 meters. Base map and data from New York City Planning [3].

Table A1. Distribution of Device Movements Three Weeks Before and Three Weeks After the Establishment of Concentric Regulatory Zones on October 9, 2020 [a]

Origin Zone	Percentage of Device Stops by Destination					Total Stops [d]
	Red	Orange	Yellow	Metro [b]	Outside [c]	
Before (September 18 – October 8, 2020)						
Red	57.2	9.8	9.4	16.8	6.8	479,899
Orange	7.6	56.7	13.1	17.7	4.9	577,847
Yellow	3.9	6.8	64.0	21.3	4.0	1,191,614
After (October 9 – October 29, 2020)						
Red	57.6	9.4	9.2	16.8	7.0	487,557
Orange	7.1	56.8	12.7	18.4	5.0	576,975
Yellow	3.6	6.5	64.8	21.2	3.9	1,184,063

a. Each mobile device movement represented a stop in a particular census block group (CBG) for >1 minute. Each such stop had an origin and a destination CBG [4]. The table accounts only for movements of devices originating in the original red, orange, and yellow containment zones, where CBGs were classified according to the scheme in Fig. 3a. The percentages in each row add to 100%. The median dwell times (in minutes) for stops outside the home during September 18 – October 8 were: Red, 47; Orange, 35; and Yellow 42. The corresponding median dwell times during October 9 – 29 were: Red, 42; Orange, 29; and Yellow, 36.
b. Metro refers to destination CBGs in the remainder of the 17-county metropolitan region outside the red, orange, and yellow zones. This 17-county region included: one county in Connecticut (Fairfield); 7 counties in New Jersey (Bergen, Essex, Hudson, Middlesex, Monmouth, Passaic, Union); and 9 counties in the rest of New York City and New York state (Bronx, Kings, Nassau, New York, Queens, Richmond, Rockland, Suffolk, Westchester).
c. Outside refers to destination CBGs beyond the 17-county Metro region.
d. Total stops include all device stops originating from each zone during each 3-week period.

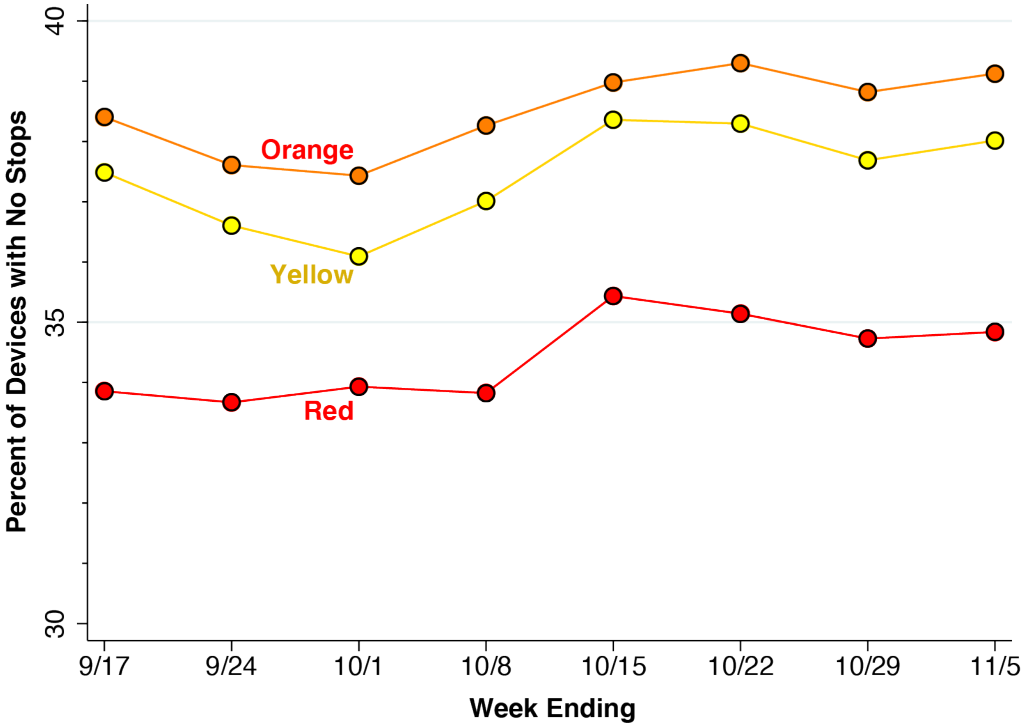

Fig. A3. Proportion of Devices Homed in Each of the Original Zones That Made No Stops During Weeks Ending 9/17 – 11/5/2020. Census block groups (CBGs) were mapped into regulatory zones in accordance with the scheme in Fig. 3a in the main manuscript. In each CBG during each day, we determined the number of devices making no stops, derived from the variable *bucketed_distance_traveled*, and the total number of devices, derived from the variable *device_count*, as recorded in the Safegraph social distancing database [4].

Appendix B: Geospatial Model

Homogeneous Mixing

An area consists of a fixed number of geographically contiguous zones, indexed $k = 1,\ldots,K$. A total of $N_k > 0$ individuals reside in zone k. At the start of each time period $t = 1,\ldots,T$, each individual residing in each zone k either remains within her zone of residence or temporarily moves to any one of the other $K - 1$ zones. At the end of time period t and before the start of time period $t + 1$, the individual returns home to zone k. Let $n_{k\ell t} \geq 0$ denote the number of movements of individuals from zone k into zone ℓ during time period t. Fig. B1 illustrates the types of movements within and between zones in a two-zone area at time t. All individuals' movements are accounted for, so that $\sum_\ell n_{k\ell t} = N_k$. Note that the number of individuals temporarily situated in zone ℓ during time period t is $M_{\ell t} = \sum_k n_{k\ell t} \geq 0$.

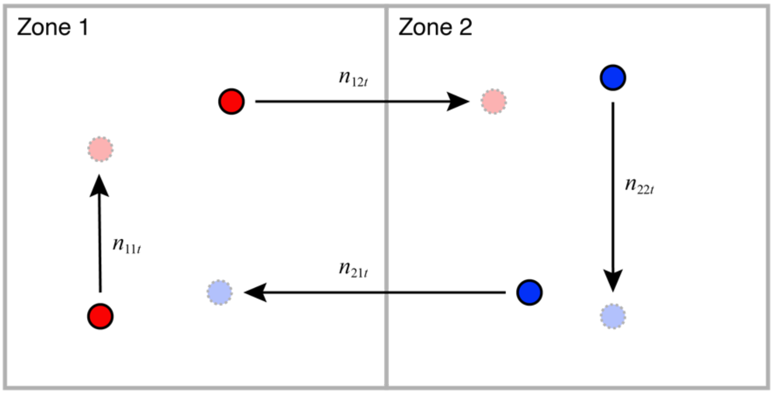

Fig. B1. Four types of movements within and between zones in a two-zone area at time t. Individuals represented by red circles reside in Zone 1 and individuals represented by blue circles reside in Zone 2. During time period t, n_{11t} residents of Zone 1 stay within their home zone, while n_{12t} residents of Zone 1 temporarily move to Zone 2. Similarly, n_{22t} residents of Zone 2 stay within their home zone, while n_{21t} residents of Zone 2 temporarily move to Zone 1. During period t, a total of $M_{2t} = n_{12t} + n_{22t}$ individuals are temporarily situated in Zone 2. Of these M_{2t} individuals, the proportions originating from Zones 1 and 2, respectively, are $w_{12t} = n_{12t}/M_{2t}$ and $w_{22t} = n_{22t}/M_{2t}$. A total of $N_1 = n_{11t} + n_{12t}$ individuals permanently reside in Zone 1. Of these N_1 individuals, the proportions moving to Zones 1 and 2, respectively, are $v_{11t} = n_{11t}/N_1$ and $v_{12t} = n_{12t}/N_1$.

An infectious disease is spread though contact between individuals temporarily situated within the same zone. The disease has a latency, an incubation interval, and a duration of infectivity of exactly one time period. That is, an individual infected through contact with an infectious person during time period $t - 1$ is diagnosed with the disease and herself becomes infectious to others only during time period t.

We keep track of the disease according to individuals' zone of residence k and date of diagnosis t. We let $y_{kt} \geq 0$ denote the fraction of residents of zone k who were diagnosed during time period t. We refer to these quantities as the incidence of the disease. The initial incidence y_{k1} thus represents the fraction of individuals residing in zone k who are diagnosed with the disease and are infectious to others during time period $t = 1$. Except for these initially infectious individuals, the remainder of the population is susceptible to infection. We assume $y_{k1} \ll 1$, so that the initial proportion of susceptible individuals in each zone k is approximately equal to 1.

We further assume homogeneous mixing of infectious and susceptible individuals. Let $w_{k\ell t}= n_{k\ell t}/M_{\ell t}$ represent the fraction of all individuals temporarily situated in zone ℓ during time period t who reside in zone k. Then the conditional probability of infection of a randomly selected, susceptible person temporarily situated in zone ℓ during time period t is $p_{\ell t} = \alpha \sum_r w_{r\ell t} y_{rt}$, where $\alpha > 0$ is the reproductive number, an indicator of the efficiency of transmission. (In this expression, we have used the summation index r for zones of residence, rather than k.)

Now consider a randomly selected, susceptible individual residing in zone k. The probability that this individual will be temporarily situated in zone ℓ during time period t is $v_{k\ell t} = n_{k\ell t}/N_k$. Hence, the unconditional probability that this resident of zone k will be infected via contact with an infectious person during time period t is $\sum_\ell v_{k\ell t}\, p_{\ell t}$ or equivalently, $\sum_\ell (v_{k\ell t})\,(\alpha \sum_r w_{r\ell t} y_{rt})$. We thus have the dynamic equation of motion of the incidence of infection in each zone in each time period:

$$y_{k,t+1} = \alpha \sum_\ell \sum_r v_{k\ell t} w_{r\ell t} y_{rt} \qquad \text{(B1)}$$

Equation (B1) is a linear system of difference equations, in which the incidence of new cases $y_{k,t+1}$ diagnosed during time period $t + 1$ among residents of zone k depends on the incidence $\{y_{rt}\}$ of cases previously diagnosed at time t in all zones $r = 1,\ldots,K$. It is an adaptation of the conventional law of mass action implicit in SIR-type compartmental models of the dynamics of contagious disease transmission [5]. Fig. B2 illustrates the three ways that a susceptible person can become infected in a two-zone model during a particular time period.

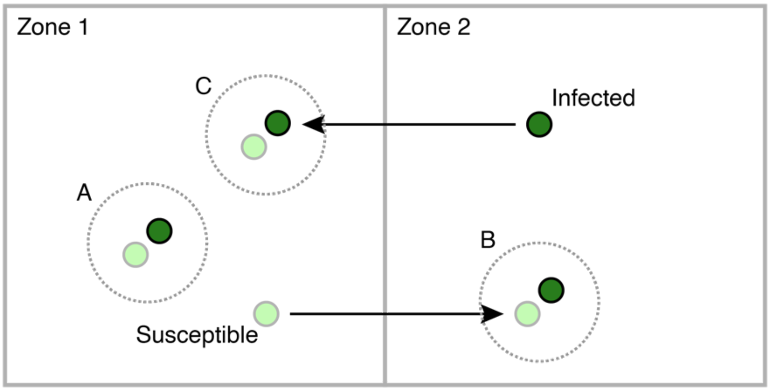

Fig. B2. Three ways that a susceptible individual can become infected in a two-zone model during a particular time period. In case A, the susceptible individual comes into contact with an infected individual who is also a resident of Zone 1. In case B, the susceptible individual temporarily moves to Zone 2 and comes into contact with an infected individual who resides in Zone 2. In Case C, the susceptible individual comes into contact with an infected resident of Zone 2 who has temporarily moved to Zone 1.

It will be useful to convert equation (B1) into matrix notation. For each time period t, let V_t be the $K \times K$ square matrix with typical element $v_{k\ell t}= n_{k\ell t}/N_t$, which, as noted above, represents the fraction of all residents of zone k who temporarily move to zone ℓ during time period t. The elements in the rows of V_t sum to 1. Let W_t be the $K \times K$ square matrix with typical element $w_{k\ell t}= n_{k\ell t}/M_{\ell t}$, which, as noted above, represents the fraction of all individuals temporarily situated in zone ℓ during time period t who reside in zone k. The elements of the columns of W_t sum to 1. Finally, let Y_t denote the $K \times 1$ column vector $(y_{1t}, \dots, y_{Kt})'$. Then our dynamic model can be represented in matrix form as:

$$Y_{t+1} = \alpha\, V_t\, W_t{}'\, Y_t \qquad \text{(B2)}$$

In equation (B2), all the elements of the $K \times 1$ column vector $V_t\, W_t{}'\, Y_t$ are multiplied by the same scalar parameter α. If we already have data on the movements $\{n_{k\ell t}\}$ between zones and data on the incidence $\{y_{kt}\}$ of the disease, then the matrices V_t and W_t and the vectors Y_t are likewise observed quantities, and thus we can estimate the reproductive number α as the only unknown parameter.

Inhomogeneous Mixing

We consider two modifications of equation (B2) to capture inhomogeneous mixing [6]. First, movements of individuals who remain within their zone of residence may have a lower reproductive number α_0. Second, movements of individuals who reside in or enter certain high-

risk zones may have a higher reproductive number α_1. All other movements are assumed to have the same reproductive number α_2.

To capture within-zone movements, let D_t = diag($V_t W_t{}'$) denote the $K \times K$ square matrix with the same diagonal elements as $V_t W_t{}'$ but zero off-diagonal elements. Define the $K \times K$ square matrix $X_t = V_t W_t{}' - D_t$, that is, the matrix $V_t W_t{}'$ with its diagonal elements set to 0. Then our model becomes:

$$Y_{t+1} = \alpha_0 D_t Y_t + \alpha_1 X_t Y_t \qquad \text{(B3)}$$

To further capture differences in transmission efficiency between zones, we partition the set of zones into two nonempty subsets, L and H, representing the low- and high-transmission zones, respectively. The former has K_L elements and the latter has $K_H = K - K_L$ elements. We assume all zones in L have the same reproductive number α_1, while all zones in H have reproductive number α_2. Partition the square $K \times K$ matrix X_t vertically into two matrices: a $K \times K_L$ matrix X_{tL}, and a $K \times K_H$ matrix X_{tH}. Similarly, partition the column vector Y_t horizontally into two vectors: Y_{tL} and Y_{tH}. Then equation (B3) becomes:

$$Y_{t+1} = \alpha_0 D_t Y_t + \alpha_1 X_{tL} Y_{tL} + \alpha_2 X_{tH} Y_{tH} \qquad \text{(B4)}$$

Model Implementation

The area under study consists of K = 21 ZCTAs situated in South Brooklyn, as shown in Fig. 2 of the main text. We focus on the 7-week period running from the week ending October 17 through the week ending November 28, that is, the last 7 images in Fig. 6 in the main text. We derive the incidence y_{kt} for k = 1,...,21 and t = 1,...,7 from confirmed COVID-19 cases per 100,000 population, as reported by the New York City Department of Health [7].

We relied upon the variables variables *origin_census_block_group* and *destination_cbgs* in the Safegraph Social Distancing database [4] to identify all device movements between census block groups (CBGs) within the 21-ZCTA area. Assigning each CBG to a specific ZCTA based upon the location of its centroid, we were thus able to compile data $n_{k\ell t}$, representing the number of device movements from ZCTA k to ZCTA ℓ during week t. We further relied on the variable *bucketed_distance_traveled* to count the number of devices that made no movements in each ZCTA k during each week t, and then added these counts to the within-ZCTA movements n_{kkt}. While we also observed device movements beyond the 21-ZCTA regulated area, as well as movements into the regulated area from outside, we focused sharply on the regulated area in

order to ascertain how the traffic between local ZCTAs influenced the dynamics of COVID-19 transmission.

Given the incidence data $\{y_{kt}\}$ and movement counts $\{n_{k\ell t}\}$, we estimated the models of equations (B2), (B3) and (B4) by weighted least squares, where the weights were the populations of each ZCTA. The results are displayed in Appendix C, Table C1.

Inclusion of Potentially Confounding Factors

While our geospatial model specifically addresses the influence of within- and between-ZCTA movements on COVID-19 incidence, it does not thus far account for potential confounding factors. As portrayed in Fig. B2, all new incident infections are mediated through mixing of susceptible and infected individuals. Accordingly, any relevant differences among ZCTAs in their demographic or other characteristics would need to be incorporated as multipliers to the mixing matrices $V_t W_t{}'$. More specifically, let Z denote a $K \times 1$ vector of a specific time-independent characteristic, such as the proportion of black, non-Hispanic persons, where element z_k is the proportion of such persons in ZCTA k. Then we could specify an extended version of equation (B2) as

$$Y_{t+1} = \alpha \, V_t \, W_t' Y_t + \beta Z' V_t \, W_t' Y_t \qquad \text{(B5)}$$

The parameter β would gauge the average effect of the proportion of black, non-Hispanic persons on the overall reproductive number. Similarly, equation (B3) would be extended to

$$Y_{t+1} = \alpha_0 \, D_t \, Y_t + \alpha_1 \, X_t \, Y_t + \beta_0 \, Z' D_t \, Y_t + \beta_1 Z' X_t Y_t \qquad \text{(B6)}$$

Equation (B4) would be extended to

$$Y_{t+1} = \alpha_0 \, D_t \, Y_t + \alpha_1 X_{tL} Y_{tL} + \alpha_2 X_{tH} Y_{tH} + \beta_0 \, Z' D_t \, Y_t + \beta_1 Z' X_{tL} Y_{tL} + \beta_2 Z' X_{tH} Y_{tH} \qquad \text{(B7)}$$

To test the influence of potential confounders, we merged our model database with ZCTA-specific information on three demographic characteristics derived from NYC Open Data [8]: the proportion of the population identified as Hispanic Latino, the proportion black non-Hispanic, and the proportion receiving public assistance. These three characteristics showed significant variation among the 21 ZCTAs in our database. Unfortunately, other data from the New York City Environmental & Health Data Portal [9] were keyed to a neighborhood-specific geographic classification scheme distinct from the ZCTAs used by the New York City Department of Health.

The results are displayed in Appendix C, Table C2. None of the coefficients of these covariates achieved statistically significance at the 5-percent level. The estimate for α_2 in Model B1 increased to 2.917 with 95% confidence interval [1.525, 4.370], while the corresponding estimate for α_2 in Model B2 increased to 1.813 with 95% confidence interval [1.079, 2.546]. The latter estimate was still not significantly different from α_0 (p = 0.210) or from α_1 (p = 0.089).

Appendix C

Table C1. Parameter Estimates for Geospatial Models with Homogeneous and Inhomogeneous Mixing. [a]

Parameter [b]	*Homogeneous Mixing, Eq. (2)* [c]	*Inhomogeneous Mixing, Eq. (3)* [c]		
		Model A [d]	*Model B1* [d]	*Model B2* [d]
α	**1.157** (0.026)			
α_0		1.112 (0.118)	1.099 (0.115)	1.120 (0.117)
α_1		1.228 (0.187)	0.846 (0.236)	0.735 (0.369)
α_2			**1.910** (0.324)	1.485 (0.249)
R^2 statistic	0.942	0.942	0.945	0.943
Root MSE	23.846	23.928	23.416	23.794
p-value [d] $H_0: \alpha_0 = \alpha_1$		0.699		
p-value [d] $H_0: \alpha_1 = \alpha_2$			0.012	0.124

a. All models had 126 observations. Root MSE = Root mean squared error.
b. Parameters described in equations (2), (3) and (4) in main text and in equations (B2) – (B4) above.
c. Standard error is shown below each parameter estimate. Estimate is shown in bold-face if two-sided null hypothesis that parameter = 1 is rejected (based upon F-test) at $p < 0.05$.
d. Model A distinguished between within-ZCTA effects (α_0) and all other effects (α_1). Models B1 and B2 distinguished between within-ZCTA effects (α_0), other low-risk effects (α_1), and other high-risk effects (α_2). Model B1 was based on the classification of five ZCTAs (11223, 11229, 11235, 11224, and 11214) as high risk, while Model B2 was based upon classification of all original red-zone ZCTAs as high risk.
e. Other tests of two-sided null hypotheses (H_0) based upon F-tests.

Table C2. Parameter Estimates for Geospatial Models with Homogeneous and Inhomogeneous Mixing, Including Demographic Covariates. [a]

Parameter [b]	*Homogeneous Mixing, Eq. (2)* [c]	*Inhomogeneous Mixing, Eq. (3)* [c]		
		Model A [d]	*Model B1* [d]	*Model B2* [d]
α	**1.228** (0.041)			
α_0		1.182 (0.139)	1.172 (0.137)	1.258 (0.143)
α_1		1.336 (0.234)	0.499 (0.396)	0.269 (0.656)
α_2			**2.917** (0.703)	**1.813** (0.370)
β (Hispanic)	–0.071 (0.250)			
β_0 (Hispanic)		–0.791 (2.192)	1.719 (2.410)	–1.403 (2.283)
β_1 (Hispanic)		1.095 (3.304)	2.291 (3.433)	9.805 (6.740)
β_2 (Hispanic)			–9.009 (7.358)	–3.113 (4.261)
β (Black)	–0.290 (0.164)			
β_0 (Black)		–1.158 (1.433)	–1.595 (1.823)	–1.631 (1.824)
β_1 (Black)		0.786 (1.992)	2.310 (2.924)	2.317 (3.247)
β_2 (Black)			1.600 (2.618)	1.322 (2.272)
β (Assistance)	–0.188 (0.129)			
β_0 (Assistance)		–0.491 (0.648)	–0.592 (0.642)	–0.962 (0.732)
β_1 (Assistance)		0.179 (0.987)	2.310 (1.436)	1.508 (2.144)
β_2 (Assistance)			–1.901 (2.899)	0.364 (2.063)
R^2 statistic	0.945	0.946	0.950	0.948
Root MSE	23.523	23.679	23.201	23.549
p-value [d] $H_0: \alpha_0 = \alpha_1$		0.671		
p-value [d] $H_0: \alpha_1 = \alpha_2$			0.015	0.089

a. All models had 126 observations. Root MSE = Root mean squared error.

b. Parameters described in equations (B5) – (B7) above. The three demographic covariates were: proportion Hispanic Latino; proportion black non-Hispanic; and proportion receiving public assistance.
c. Standard error is shown below each parameter estimate. Estimate is shown in bold-face if two-sided null hypothesis that parameter = 1 is rejected (based upon F-test) at p < 0.05.
d. Model A distinguished between within-ZCTA effects (α_0) and all other effects (α_1). Models B1 and B2 distinguished between within-ZCTA effects (α_0), other low-risk effects (α_1), and other high-risk effects (α_2). Model B1 was based on the classification of five ZCTAs (11223, 11229, 11235, 11224, and 11214) as high risk, while Model B2 was based upon classification of all original red-zone ZCTAs as high risk.
e. Other tests of two-sided null hypotheses (H_0) based upon F-tests.

References

1. New York Governor, *Brooklyn Large Map*. 2020, https://www.governor.ny.gov/sites/default/files/atoms/files/Brooklyn_large_map.pdf: October 6.
2. New York Governor, *Brooklyn_HiRes2.pdf (Map)*. 2020, https://www.governor.ny.gov/sites/default/files/atoms/files/Brooklyn_HiRes2.pdf: October 21.
3. New York City Planning, *ZoLa: New York City's Zoning & Land Use Map*. 2022, https://zola.planning.nyc.gov/?layer-groups=%5B%22building-footprints%22%2C%22street-centerlines%22%2C%22subway%22%5D#17.92/40.600177/-73.943332: Accessed May 12.
4. SafeGraph Inc., *Social Distancing Metrics*. 2020, https://docs.safegraph.com/docs/social-distancing-metrics: Updated August 2021.
5. Kolokolnikov, T. and D. Iron, *Law of mass action and saturation in SIR model with application to Coronavirus modelling.* Infect Dis Model, 2021. **6**: p. 91-97.
6. Tsori, Y. and R. Granek, *Epidemiological model for the inhomogeneous spatial spreading of COVID-19 and other diseases.* PLoS One, 2021. **16**(2): p. e0246056.
7. New York City Department of Health and Mental Hygiene, *caserate-by-modzcta.csv (Weeks ending 8/8 through 11/28/2020)*. 2020, https://raw.githubusercontent.com/nychealth/coronavirus-data/84dead220def7b53bcb2d9f9210609dee9446045/trends/caserate-by-modzcta.csv: Updated December 9.
8. NYC OpenData, *Demographic Statistics By Zip Code*. 2022, https://data.cityofnewyork.us/City-Government/Demographic-Statistics-By-Zip-Code/kku6-nxdu: New York City Department of Youth and Community Development, May 9.
9. City of New York, *Environment & Health Data Portal*. 2022, https://a816-dohbesp.nyc.gov/IndicatorPublic/Subtopic.aspx: Accessed September 29.
